# Dry powder formulations of hyperimmune serum

**DOI:** 10.1007/s13346-024-01678-8

**Published:** 2024-07-31

**Authors:** Annalisa Bianchera, Gaetano Donofrio, Fabio Sonvico, Ruggero Bettini

**Affiliations:** 1 Department of Food and Drug Sciences, University of Parma, Parco Area Delle Scienze 27/a, 43124 Parma, Italy; 2 Interdepartmental Research Centre for the Innovation of Health Products, University of Parma, Parco Area Delle Scienze, Biopharmanet-TecPadiglione 33, 43124 Parma, Italy; 3 Department of Medical-Veterinary Science, University of Parma, Via del Taglio 10, 43126 Parma, Italy

**Keywords:** Inhalable powders, Hyperimmune serum, Immunoglobulins formulation, Viral neutralization, Prophylaxis

## Abstract

Effective strategies against the spread of respiratory viruses are needed, as tragically demonstrated during the COVID-19 pandemic. Apart from vaccines, other preventive or protective measures are necessary: one promising strategy involves the nasal delivery of preventive or protective agents, targeting the site of initial infection. Harnessing the immune system’s ability to produce specific antibodies, a hyperimmune serum, collected from an individual vaccinated against SARS-CoV-2, was formulated as a dry powder for nasal administration. The selection of adequate excipients and process are key to maintaining protein stability and modulating the aerodynamic properties of the powders for reaching the desired respiratory regions. To this end, a hyperimmune serum was formulated with trehalose and mannitol as bulking agents during spray drying, then the ability of the redissolved immunoglobulins to bind Spike protein was verified by ELISA; foetal bovine serum was formulated in the same conditions as a reference. Moreover, a seroneutralization assay against SARS-CoV-2 pseudoviruses generated from different variants of concern was performed. The neutralizing ability of the serum was slightly reduced with respect to the starting serum when trehalose was used as a bulking agent. The powders were loaded in hypromellose capsules and aerosolized employing a nasal insufflator in an in vitro model of the nasal cavity connected to a Next Generation Impactor. The analysis of the powder distribution confirmed that all powders were inhalable and could target, at the same time, the upper and the lower airways. This is a preliminary proof-of-concept that this approach can constitute an effective strategy to provide broad coverage and protection against SARS-CoV-2, and in general against viruses affecting the airway. According to blood availability from donors, pools of hyperimmune sera could be rapidly formulated and administered, providing a simultaneous and timely neutralization of emerging viral variants.

**Graphical Abstract:**

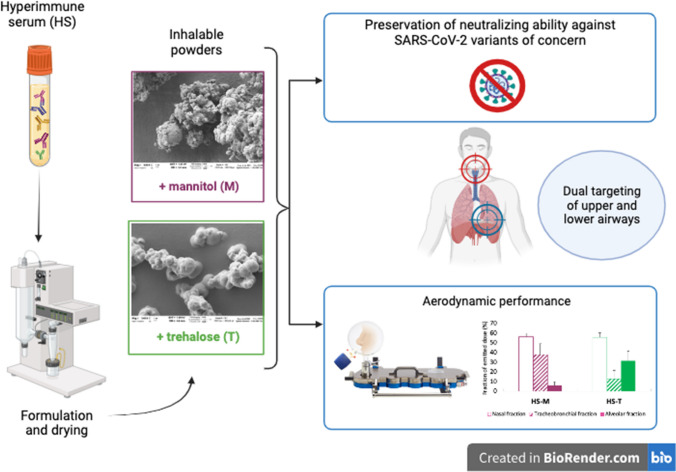

**Supplementary Information:**

The online version contains supplementary material available at 10.1007/s13346-024-01678-8.

## Introduction

The global healthcare crisis observed after the spread of Severe Acute Respiratory Syndrome Corona Virus 2 (SARS-CoV-2) in 2020 put into evidence the urgent need for rapid and efficient strategies against the diffusion of that virus as well as other respiratory pathogens. Vaccines are a powerful preventive tool to counteract viruses but, the identification and production of an efficient vaccine against new viral species is time-consuming and expensive, and must keep pace with mutational escape [1]. Furthermore, the relentless emergence of viral variants that reduce vaccine effectiveness, hampers the control of viral spread. On May 5th, 2023 WHO declared the end to the COVID-19 pandemic status, and the virus is now reaching the endemic state; however, the potential emergence of new variants of this or other viruses still constitutes a substantial global health threat. For this reason, apart from looking for effective antiviral substances, many concurrent preventive strategies are necessary and desirable to precociously contribute to viral neutralization.

A prompt strategy for the neutralization of respiratory viruses should retrace their transmission path and block the infection at its very beginning. The airways, and in particular the nose, are the main site of entry and of primary infection for SARS-CoV-2, as a consequence of the high epithelial expression of angiotensin-converting enzyme 2 (ACE2) and of the membrane-bound serine protease TMPRSS2, that allow virus interaction and internalization into epithelial cells [2]. If not controlled, the virus spreads by apical shedding in the lower airways, the lung, and ultimately systemic circulation, with a concomitant progression towards a more and more severe disease. For this reason, the nose is the most straightforward administration site to tackle SARS-CoV-2 infection. As recently reviewed, nasal delivery is already under investigation not only for treatment but also for the prevention of COVID-19 [3]. Among others, some nasal sprays containing small-molecule antiviral compounds [4], barrier polymers [5, 6], or fusion inhibitory lipopeptides [7] have been proposed as pre-exposure prophylactic products to prevent SARS-CoV-2 early infection and transmission, with good effectiveness in animal models.

Learning from nature, the most specific and long-lasting tool to block infections is the recognition and elimination of the pathogen by the immune system mediated by immunoglobulins (Igs). Neutralizing monoclonal antibodies have been proposed as therapies in severe cases of COVID-19, but, as for vaccines, their effectiveness is sometimes limited by mutational escape, especially if a single type of monoclonal antibody is used instead of a combination of two or more immunoglobulins [8]. The human immune system, when in contact with a virus, produces and naturally selects a pool of exquisitely specific antibodies, with an adequate variety to contrast mutational escape that is worth exploiting. Moreover, unlike antibodies produced by phage or yeast display, that may show poor stability and/or pharmacokinetics, B-cell secreted antibodies are already preselected for stability and activity [8]. During the most severe phase of the pandemic, hyperimmune sera from donors healed from COVID-19 were administered parenterally as a potential therapeutic tool, but their effectiveness is still a matter of debate and the treatment is not devoid of risks [9, 10]. Intravenous administration is the most used route for the preservation of the activity of macromolecules, that are highly sensitive to proteolytic degradation, however, when dealing with respiratory diseases, the amount of protein that reaches the lungs from the plasma is relatively low, especially in the upper respiratory tract, where the infection occurs and spreads in the early phases of the disease. Hence, the direct delivery of hyperimmune sera to the airways, and to the nose, could be an efficient strategy to exploit this resource providing several advantages [11]. Nasal administration would increase the local concentration of Igs with a lower dose, prolong their persistence in the lungs, and reduce their proteolytic degradation and their passive diffusion to the bloodstream, thus avoiding or limiting the risk of off-target systemic side effects [12, 13]. Last but not least, a less invasive route of administration would better meet patient compliance [14] and could allow a more prompt and diffuse use of this preventive/protective tool.

Pulmonary delivery of antibodies can be achieved by nebulizing protein solutions [15] or by producing inhalable powders. This last strategy is preferred because it guarantees longer physicochemical stability of the formulations without the need for a cold chain for distribution and storage. Spray-drying is the preferred method to produce inhalable powders, since, differently from freeze-drying, a free-flowing powder, rather than a cake, is obtained. Anyway, this technique exposes proteins to relatively high temperatures and mechanical stress, which could potentially damage their delicate structure during the drying process [16]. For this reason, proteins are formulated in the solid state in the presence of appropriate excipients, that contribute to the preservation of their structure and prolong their stability and shelf-life with respect to liquid formulations. The selection of adequate excipients is critical not only for the preservation of protein activity but also to guarantee an optimal aerodynamic performance to reach the desired regions of the respiratory system. This depends on the physicochemical properties of the particles, such as size, density, and shape, that determine their aerodynamic diameter. As a rule of thumb, inhaled particles having an aerodynamic diameter greater than 10 µm are expected to stop in the upper airways, while smaller particles, preferentially in a size range between 1 and 5 µm, can reach the lungs, while those less than 1 µm are exhaled [17, 18].

As a paramount example of a pharmaceutical strategy for a therapeutic approach consisting in the inhalation of dry powders containing sera from donors previously healed from a viral respiratory infection, in this paper, we propose a proof-of-concept for the formulation as an inhalable dry powder of a hyperimmune serum collected from an individual vaccinated against SARS-CoV-2. This powder is intended to be administered into the nose as a pre-exposure or early post-exposure prophylaxis in case of contact with a positive subject. The choice of the nasal rather than the oral inhalation route could potentially provide a dual targeting both of the high and low airways, as recently proposed for a monoclonal antibody by Seow et al*.* [19, 20]. The effect of two different bulking agents, namely trehalose and mannitol, was investigated concerning the preservation of the neutralizing ability of serum against five different SARS-CoV-2 spike pseudoviruses and the in vitro aerodynamic performance of resulting powders.

## Materials and methods

### Materials

Monobasic and dibasic potassium phosphate salts were from Merck (Darmstadt, Germany); trehalose was from A.C.E.F. (Fiorenzuola D’Arda, Italy), mannitol, Pearlitol 100 SD, was from Roquette (Lestrem, France); size 3 hypromellose capsules were QUALI-V-I inhalation from Qualicaps (Madrid, Spain) and were fitted into a Miat® nasal insufflator (MIAT S.p.A. Milan, Italy). Foetal bovine serum (FBS) was from Aurogene (Rome, Italy). Bio-Rad protein Assay was from Bio-Rad Laboratories GmbH (Munich, Germany). Ultrapure water was produced by an Arium® purification system (Sartorius, Goettingen, Germany).

### Serum collection and formulation as dry powder

Sera were collaboratively provided by the Unit of Infectious Diseases and Hepatology of the University Hospital of Parma. The study was approved by the local ethical committee (Comitato Etico Area Vasta Emilia Nord (AVEN), Italy). All participants gave written informed consent to participate in the study. Among the blood samples collected, one derived from a person immunized after vaccination against SARS-CoV-2 with one dose of Vaxzevria (Astra-Zeneca) and two doses of Comirnaty (Pfizer) was selected, for its ability to efficiently neutralize different pseudoviral preparation as previously published [21]. The coagulated sample was centrifuged at 2500 rpm to separate coagulated blood cell fraction from serum and decomplemented at 56 °C for 30 min.

The human serum (HS) (protein concentration 100 mg/mL, estimated by Bradford assay) was formulated by 50-fold dilution to have a final content of proteins of 2 mg/mL in 10 mM phosphate buffer (prepared by dissolving 2.36 g of K_2_HPO_4_ and 1.577 g of KH_2_PO_4_ in 1 L of ultrapure water and brought to a final pH of 7), to which mannitol or trehalose were added as bulking agents at a concentration of 14 mg/mL, in a 7:1 weight ratio with respect to serum proteins.

The solutions were then spray-dried with a Buchi B-290 mini spray drier (Büchi Labortechnik AG, Flawil, Switzerland) equipped with a 1 mm nozzle. Drying was performed at 130 °C inlet temperature, under a nebulization airflow of 700 L/h with an aspiration of 33 m^3^/h, and a solution feed rate of 3 mL/min. Formulations were coded as HS-M or HS-T according to the bulking agent used, namely mannitol or trehalose, respectively. Foetal bovine serum (FBS, protein concentration 70 mg/mL, estimated by Bradford assay) was formulated in the same conditions as negative control and the resulting powders with mannitol or trehalose were coded as FBS-M and FBS-T, respectively. All powders were stored in a desiccator at 4 °C.

### Thermogravimetric analysis of powders (TGA)

Thermogravimetric analysis of powders was performed using a TGA/DSC 1 STARe system (Mettler Toledo Inc., Columbus, USA) under a nitrogen flow of 80 mL/min in a temperature range between 25 and 150 °C with a heating rate of 10 K/min. The water content of the samples was estimated by the software STARe (Version 11, Mettler Toledo Inc., Columbus, USA).

### ELISA

Twenty-five μL of serum as such or 25 μL of a solution of formulated powders at 250 mg/mL in cell culture medium (resulting in a 1:4 dilution with respect to protein concentration in the starting serum) were tested at several dilutions on ELISA plate wells coated with soluble full-length Spike protein, deleted of the transmembrane domain, and expressed in HEK293T cells as previously described [22]. Antibodies detected were expressed as the optical density at 450 nm measured by a Wallac 1420 Victor 2 microplate reader (Perkin Elmer, Milan, Italy).

### SARS-CoV-2 pseudovirus generation and seroneutralization assay

Lentiviral vector-based SARS-CoV-2 spike pseudoviruses were generated as previously described [21]. Briefly, HEK293T cells were transfected in T175 cm^2^ flasks with pLV-EF1α-(turboGFP-Luc2)-WPRE transfer vector, p8.74 packaging vector, pLV-CMV-(S-ΔRS-HA)-IRES-Puro-WPRE pseudotyping vector and pREV (58 µg of total DNA) in the presence of PEI (Polysciences, Inc., Warrington, PA, USA, ratio 1:2.5 DNA/PEI) [21]. After transfection, the cells were disrupted by freeze-thawing at -80 °C, and the supernatant was clarified via centrifugation followed by filtration through 0.45 µm filters. Five different SARS-CoV-2 spike pseudoviruses were produced, displaying spike glycoproteins on their surface belonging to Wuhan-Hu-1 (B.1 Lineage; China), Alpha (B.1.1.7. Lineage; United Kingdom), Beta (B.1.351 Lineage; South Africa), Gamma (P.1 Lineage; Brazil), Delta (B.1.617.2 Lineage; India) or Omicron (B.1.1.529 Lineage; Europe) variants. Twenty-five µL of each pseudovirus preparation (corresponding to ~ 10^4^ relative luciferase units (RLUs) were added to 96-well plates (Greiner Bio-one) to which the same volume of EMEM with 10% FBS, and serum samples were added. Serum was tested as such or after redissolution of formulations at dilutions of 1:4, 1:8, 1:16, 1:32, 1:64, 1:128, 1:256, and 1:512 with respect to the original concentration of serum. A negative control was established without serum. The RLUs were compared and normalized to those derived from wells where pseudoviruses were added in the absence of sera (100%). Neutralization titer 50 (NT_50_) was expressed as the maximal dilution of sera with a signal reduction of ≥ 50%. Each serum was tested in triplicate.

### Analysis of particle size distribution

Particle size distribution was measured using a Spraytec laser diffraction system (Malvern Panalytical Ltd, Malvern, UK) equipped with a 300 mm focal lens. Ten mg of each powder were dispersed in a 0.1% Span® 85 solution in cyclohexane. The suspension was sonicated for 1 min right before the measurement, which was carried out with an obscuration threshold of at least 10%. Particle size data are presented as Dv_10_, Dv_50_, and Dv_90_, indicating equivalent volume diameters at 10%, 50%, and 90% of cumulative volume distribution, respectively. The width of the particle size distribution was expressed by Span, calculated as (Dv_90_- Dv_10_)/Dv_50_.

### SEM analysis of particles

Powder morphology was investigated with a Field Emission Scanning Electron Microscope (SEM) equipped with a Ga-Focused Ion Beam (FIB) Auriga Compact (Zeiss, Oberkochen, Germany). A few milligrams of each sample were dispersed on carbon tapes placed on aluminium stubs. The surface morphology and texture of the microparticles were investigated in plan-view by using a 1 kV electron beam acceleration voltage at a working distance of about 5 mm.

### Differential Scanning Calorimetry (DSC)

A DSC 821e (Mettler Toledo, Switzerland) driven by STARe software (Mettler Toledo) was employed to investigate the thermal behaviour of powders in the temperature range between 25 °C and 150 °C at a heating rate of 5 °C/min. The instrument was previously calibrated with Indium (onset of melting Tm = 156.48 ◦C, enthalpy of melting ∆Hm = 28.60 J g − 1).

Samples of about 8 mg were placed in a 40 μL aluminum pan with a pierced cover and heated under a flux of dry nitrogen (100 mL/min).

### Evaluation of aerosolization performance

Twenty-five mg of powders were loaded in size 3 hypromellose capsules and placed in a Miat® nasal insufflator. The accurate amount of powder in each capsule was recorded. The capsule was pierced, and the nasal adapter of the insufflator was inserted into the hole of a nasal expansion chamber (volume 2 L) mounted on a Next Generation Impactor (NGI, Copley Scientific, Nottingham, UK) connected to a vacuum pump (VP 1000, Erweka GmbH, Germany) set to generate an aspiration flow of 15 L/min. The flow rate and time were controlled by a Critical Flow Controller (TPK, Copley Scientific, Nottingham, UK) at 15 L/min for 15 s. Five seconds after activating the pump, the rubber bulb of the insufflator was squeezed to aerosolize the content of the capsule while aspiration was maintained for 10 s. The mass distribution of emitted proteins was assessed by quantitatively collecting the powder with ultrapure water in the nasal expansion chamber, in the stages of the impactor, and on the filter of the Micro Orifice Collector. Each sample was brought to volume in volumetric flasks and protein concentration was quantified by Bradford protein assay [23] as by producer’s specifications for the microplate assay. The amount of powder loaded in the capsule was based on preliminary tests performed to guarantee that the sensitivity of the analytical method was enough to detect the protein distributed in all stages of the NGI apparatus. The absorbance of samples at 595 nm was measured by a Spark 10 M plate reader (Tecan, Männedorf, Switzerland), and protein concentration was determined with respect to a calibration curve prepared with bovine serum albumin. The amount of protein collected in the nasal expansion chamber was considered separately and coded as nasal fraction (NF), while the cumulative undersize mass percentage of proteins found in each stage was used to build a mass distribution plot with respect to the cut-off diameters of each stage: in particular, the median mass aerodynamic diameter (MMAD) and the geometric standard deviation (GSD) were calculated from the plot of cumulative undersize percentage of the collected drug (in probit scale) with respect to the log cut-off values of each stage at a flow of 15 L/min according to European Pharmacopoeia specifications (2.9.18, 10th Ed.) [24]; the plot was built with Excel for Mac (ver. 16.57, Microsoft Corp., Redmond, USA). This plot also allowed the determination of the respirable fraction (RF%), corresponding to the percentage mass of protein emitted as powders having an aerodynamic diameter lower than 5 μm. A single capsule was discharged for each test, which was repeated three times.

## Statistics

Data were analysed with Microsoft Excel for Mac (v. 16.83) and presented as a mean plus standard deviation; data were compared by student's *t-test*. Graphs were built with KaleidaGraph v. 4.5.4 (Synergy Software, Reading, USA).

## Results

### Spray-drying yield and water content of powders

The yield of the spray drying process was quite low (36%) for the mannitol-based serum formulation (HS-M) and satisfactory (83%) for the trehalose-based serum formulation (HS-T). Drying of FBS-M and FBS-T gave a yield of 65% and 68%, respectively. The protein content was estimated by Bradford assay at 16% w/w for HS-based formulations and 10% w/w for FBS-based formulations, with respect to the nominal amount of 15% w/w and 7.2% w/w, respectively. Water content, as estimated by TGA, was comparable for the powders, being 4.4 ± 0.2% for HS-M and 6.2 ± 1.7% for HS-T, while For FBS-M water content was 4.0 ± 0.5% and for FBS-T 6.4 ± 1.8%. The protein content was checked confirming that the composition of the spray-dried powder did not change significantly, being 16% w/w for HS-based formulation with respect to a nominal amount of 15% w/w, and 10% w/w for FBS with respect to a nominal amount of 7.2% w/w.

### Binding to spike protein

**Fig. 1:**
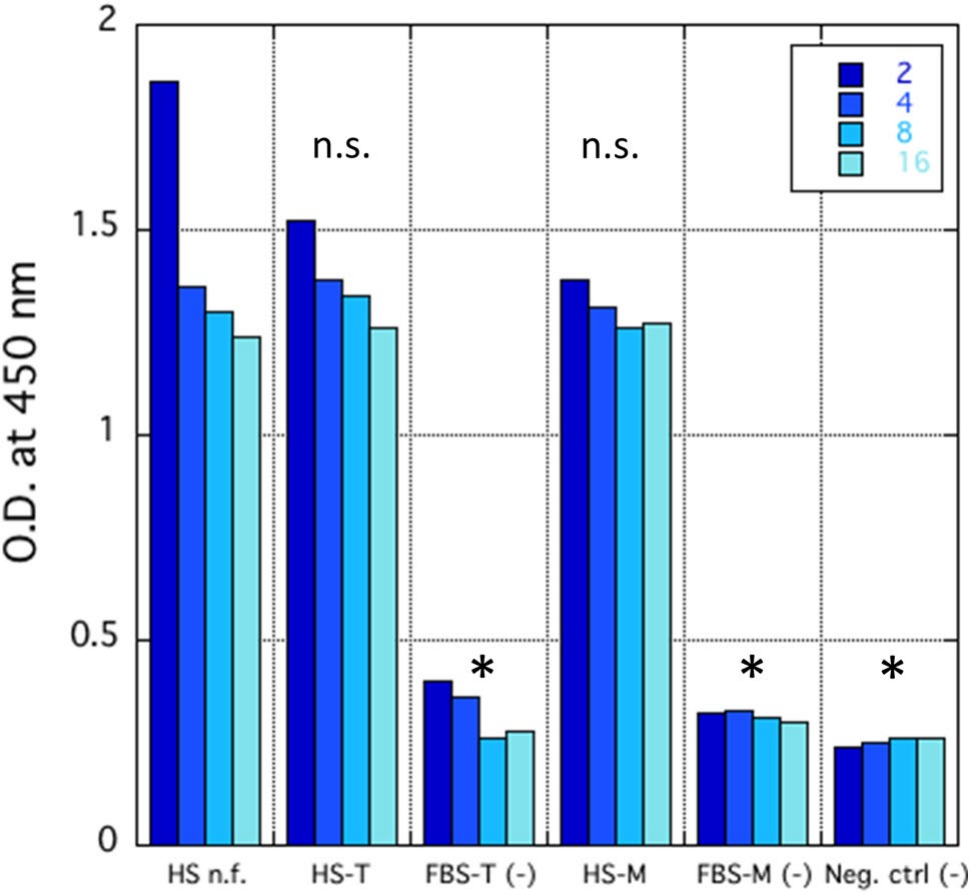
Optical densities obtained from ELISA against Spike protein of serum as such or reconstituted from formulated powders. Bar colours indicate sera dilutions (2, 4, 8, or 16 times). HS = hyperimmune serum, FBS = foetal bovine serum, n.f. = not formulated, T = trehalose, M = mannitol, neg.ctrl. = negative control. * = significant difference with respect to HS n.f. (p < 0.001); n.s. = no significant difference with respect to HS n.f

Drying can potentially modify the conformation of antibodies and, consequently, their affinity for the antigen. An ELISA was performed to estimate the ability to bind the Spike protein of the prepared serum-containing powders. Each serum was tested in triplicate, giving an identical O.D., therefore no standard error was obtained. Each condition was compared with serum as such, with the corresponding formulation of FBS and with the negative control using the student's *t-test*. 0.5 O.D. was assessed as a stringent threshold of positivity. Figure 1 shows that the binding ability of serum after the redissolution of both powders was comparable to that of serum before formulation and that no interference in the assay was derived from excipients, as witnessed by the low binding abilities of FBS-based formulations that were comparable to negative control.

### Neutralizing ability

**Fig. 2:**
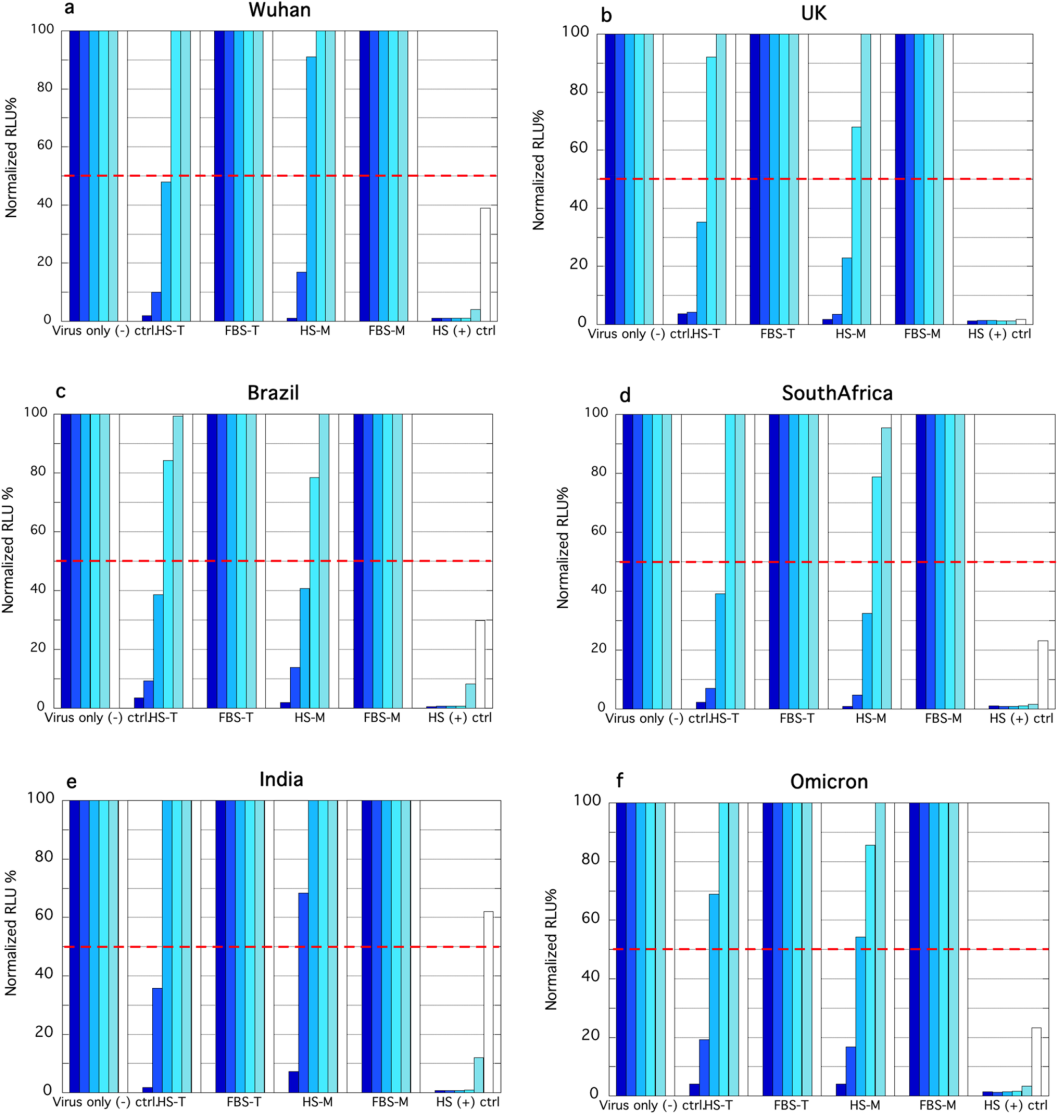
Neutralization ability of serum as such or reconstituted from formulated powders represented as reduction of luciferase activity by SARS-CoV-2 pseudoviral preparations representative of variants: **a**) Wuhan **b**) Alpha, UK **c**) Beta, Brazil **d**) Gamma, South Africa **e**) Delta, India **f**) Omicron. Bar colours indicate sera dilutions (from dark blue to white: 16, 32, 64, 128, 256, or 512 times). HS = hyperimmune serum, FBS = foetal bovine serum, n.f. = not formulated, T = trehalose, M = mannitol. The dashed red line highlights the NT_50_ value (n = 3)

Afterward, the neutralizing effect of powders was assessed and compared to the unformulated serum. The neutralization assay using pseudotyped viruses is worldwide considered a convenient tool to analyse immune responses, and applied to the screening of commercial antibodies [25] since a high level of neutralizing antibodies is reported to be correlated to protection against viral infections [26–28] by preventing the penetration of viruses. After their redissolution in culture medium, the neutralization titre of each powder against 6 different SARS-CoV-2 pseudoviral preparations representative of as many variants of concern was tested as reported in Fig. 2.

The normalized NT_50_/mL is summarised in Table 1.

**Table 1:** Normalized NT_50_/mL of serum as such or upon formulation with different bulking agents (HS-T = trehalose, HS-M = mannitol)

SARS-CoV-2 Variant	Serum	HS-T	HS-M
Wuhan	1:20480	1:10240	1:5120
UK	1:20480	1:10240	1:10240
Brazil	1:20480	1:10240	1:10240
South Africa	1:20480	1:10240	1:10240
India	1:10240	1:5120	1:2560
Omicron	1:20480	1:5120	1:5120

The results confirm the ability of the starting serum to efficiently neutralize all pseudoviral preparations. Drying induced only a slight decrease in the neutralizing ability of serum, independent of the bulking agent used, as assessed by the comparable values of NT_50_/mL. Trehalose was more effective in preserving the neutralizing ability compared to mannitol if Wuhan and India pseudoviral variants are considered. We ascribe the differences in neutralization to a different ability of the excipients to preserve antibody configuration during the drying process, and not to an interference due to the nature of the excipients used. In fact, as reported by Hufnagel et al*.* [29] and Arte et al*.* [30], the activity of monoclonal antibodies is not modified by the simple presence of these excipients in solution, and only the appearance of protein microaggregates was reported.

## Characterization of powders and aerosolization performances

### Particle size distribution analysis

The results of particle size distribution analysis are reported in Table 2. The corresponding values referred to FBS-T and FBS-M are reported in Table S1 in Supplementary materials.

**Table 2:** Particle size distribution of human serum-containing powders formulated with trehalose (HS-T) or mannitol (HS-M) as bulking agents

Formulation	D_V(10)_ μm	D_V(50)_ μm	D_V(90)_ μm
HS-T	1.97 ± 0.02	4.90 ± 0.12	12.65 ± 39.09
HS-M	2.57 ± 0.02	10.01 ± 0.07	21.73 ± 0.65

**Fig. 3:**
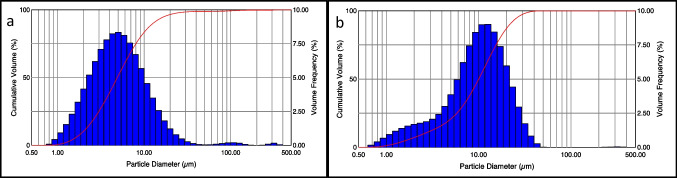
Particle size distribution as volume frequency (blue bars) and cumulative volume distribution (red line) of (**a**) HS-T and (**b**) HS-M

As can be better appreciated in Fig. 3a, HS-T showed a monomodal distribution with a Dv_50_ close to 5 µm. On the other hand, HS-M (Fig. 3b) showed a bimodal particle size distribution, with a Dv_50_ close to 10 µm but left-skewed, for the presence of smaller particles. The particle size distribution was adequate for inhalation, with the potential to impact in the nasal cavity (for particles above 10 µm) and to reach the lower airways. Similar results were obtained for FBS-T and FBS-M and the relevant graphs are reported in Figure S1.

### SEM analysis

**Fig. 4:**
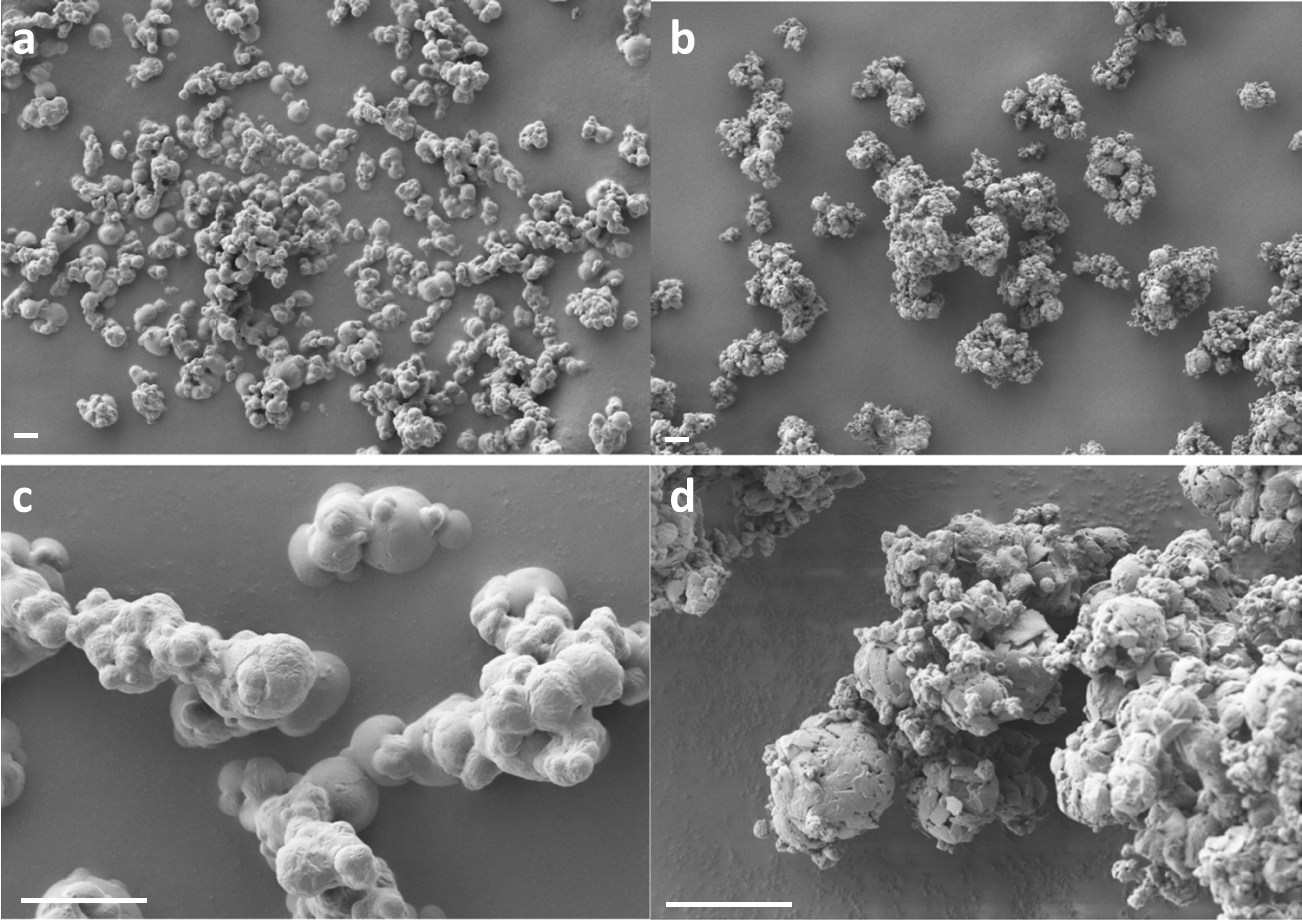
SEM images of HS-T (**a**-**c**) and HS-M (**b**-**d**), magnification 1000 X (**a**-**b**) and 5000 X (**c**-**d**). White bars in the bottom left correspond to 10 µm in length

SEM analysis of samples (Fig. 4 and Figure S2) revealed a different appearance for the powders prepared with trehalose or mannitol as bulking agents. All formulations showed the presence of aggregates of smaller particles, but when trehalose was used, aggregates appeared smoother, as if particles were embedded and entrapped in a fused matrix. This behaviour could be attributed to the formation of water bridges between amorphous trehalose particles, as previously observed [31]. On the other hand, aggregates produced with mannitol were bigger and composed of larger and smaller particles, the former having a rough surface, scales, and cracks, and the latter appearing as loosely bound aggregates. These data agree with results obtained by laser diffraction analysis and can explain the bimodal distribution observed in HS-M formulation, which can be probably due to the detachment of smaller particles from larger aggregates.

**Fig. 5:**
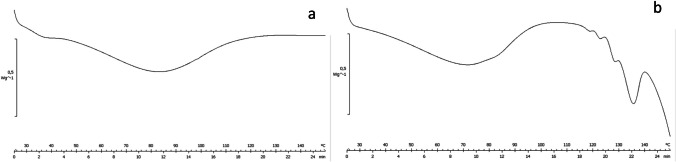
DSC traces of HS-T (**a**) and HS-M (**b**)

This different appearance can be attributed to the different glass transition temperatures (T_g_) of the two bulking agents. Trehalose has a T_g_ of 117 °C and forms an amorphous glass matrix after spray drying. On the other hand, mannitol has a low T_g_ of 11 °C and recrystallises after spray-drying [32]. The evaluation by DSC (Fig. 5 and Figure S3) of samples confirms that the trehalose-based formulations are in amorphous form (even after 2 years of storage at 4 °C), while the mannitol-based ones show some signs of crystallization in the form of δ-mannitol as revealed by the first melting peak at 129 °C [33].

### Assessment of aerodynamic behaviour by NGI

The powder emitted by the Miat® (Milan, Italy) nasal insufflator, independently of the bulking agent used, was comparable, with an emitted protein fraction of 56 ± 15% for HS-T and 56 ± 5% for HS-M. The emitted protein fraction was 74 ± 27% for FBS-T and 83 ± 12% for FBS-M. The powder was mainly recovered in the expansion chamber, accounting for 64 ± 1% and 58 ± 7% of the emitted dose for HS-T and HS-M, respectively. Analogously, the percentage of powder trapped in the expansion chamber was 73 ± 9% for FBS-T and 84 ± 12% for FBS-M. When mannitol was used, most of the powder was collected in the first 4 stages of the NGI, affording a MMAD of 24 ± 1 μm, and a respirable dose of proteins of 0.2 ± 0.1 mg. On the other hand, trehalose-based formulations reached lower stages of the impactor, with a MMAD of 4.60 ± 1.81 μm and a higher respirable dose (0.7 ± 0.2 mg), suggesting that this formulation could also reach the deep airways and potentially exert its neutralizing effect against SARS-CoV-2 both in the nose and in the lungs (Table 3 and Table S2).

**Table 3:** Distribution in the NGI of hyperimmune serum formulated with trehalose (HS-T) or mannitol (HS-M) as bulking agents

	HS-T	HS-M
Emitted dose of protein (mg)	2.6 ± 0.7	2.8 ± 0.2
Nasal dose of protein (mg)	1.5 ± 0.5	1.6 ± 0.1
Trachea + lung dose of protein (mg)	1.0 ± 0.4	1.2 ± 0.4

**Fig. 6:**
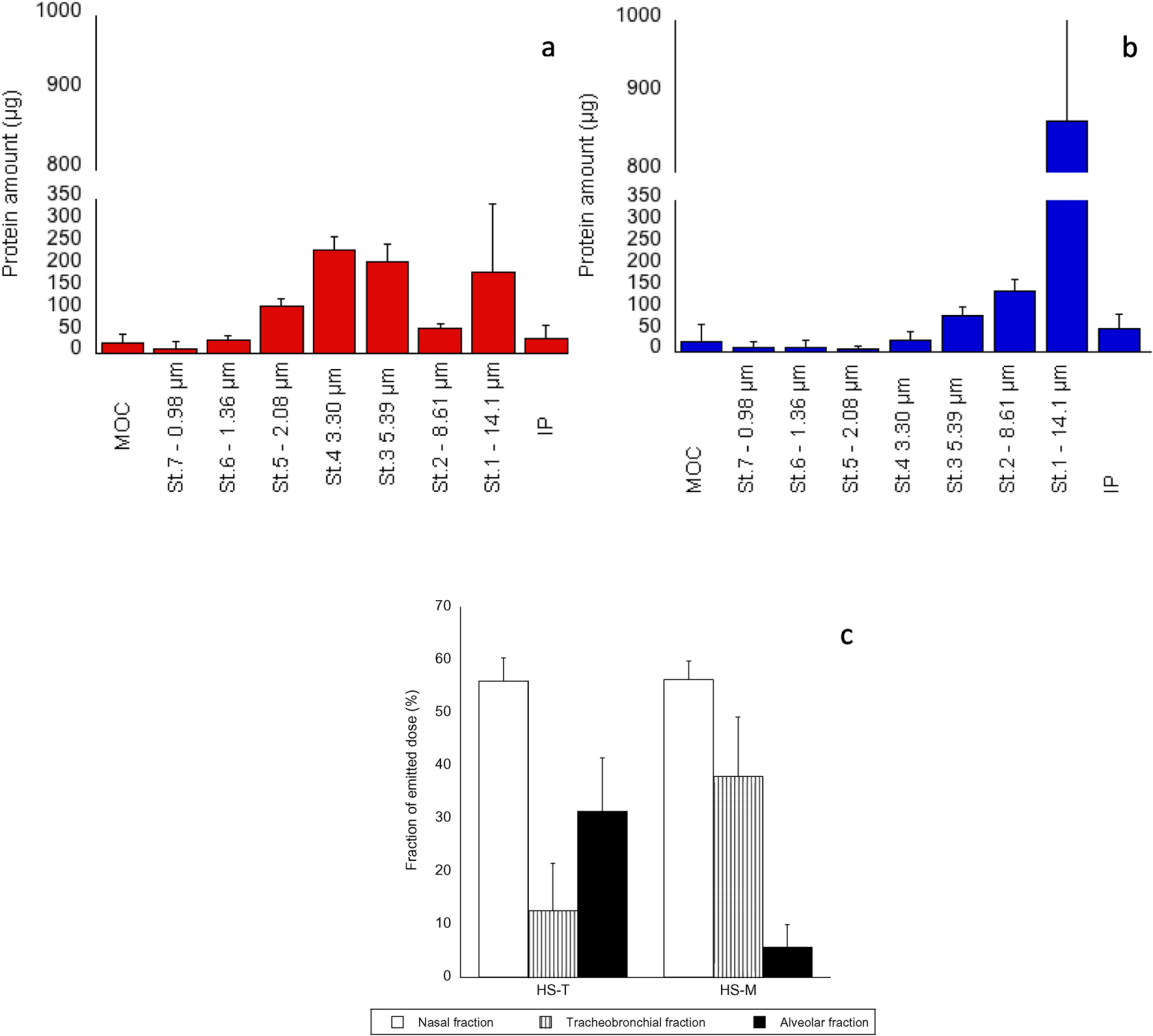
Aerodynamic distribution of HS-T (panel **a**) and HS-M (panel **b**) in the stages of NGI. Fractions of formulations HS-T and HS-M supposed to reach the nose (empty column), tracheobronchial area (striped column), and alveoli (full column) after nasal inhalation (panel **c**). The bars represent the standard deviation (n = 3)

Figure 6 shows the aerodynamic distribution of the mannitol-containing powder (panel a) and the trehalose–containing powder (panel b) as well as the relevant comparison of the fractions of powders impacting in the expansion chamber, coded as nasal fraction, the upper airways (d_ae_ above 5 µm, corresponding to IP fraction + stages 1 and 2) coded as tracheobronchial fraction, and alveoli (d_ae_ below 5 µm). Analogous results in terms of aerodynamic distribution were obtained for FBS-T and FBS-M, as reported in Figure S4 a, b, and c.

## Discussion

The topical administration by nebulization of human plasma-derived immunoglobulins to rats and non-human primates has already proven effective against acute respiratory infections [15]. However, the stability of proteins in solution is generally short, and a solid formulation would be desirable, provided that the neutralization ability of Igs is preserved during the drying process [34]. In this paper, we present a proof of concept for the formulation of a hyperimmune serum as inhalable powders to be administered into the nose as a pre-exposure or early postexposure prophylaxis to viral infection, with the purpose of locally neutralizing the viral particles at the very early stage of contact with airways. Because the COVID-19 pandemic has generated a lot of interest in terms of vaccination strategies against agents inducing lung pathology, and several laboratory models have been established [35], SARS-CoV-2 was chosen as a model to test the biological stability of the dry powder formulations of hyperimmune serum. Before formulation, the ability of serum to efficiently bind a recombinant version of Spike protein and to neutralize in vitro different variants of concern of SARS-CoV-2 was verified. To obtain a respirable powder, spray drying was chosen as the preparation method [36], starting from the hyperimmune serum diluted in a 10 mM potassium phosphate buffer (pH 7): the choice of this buffer was based on previous reports describing the ability of PBS in stabilizing pH and in preserving the potency of a measles vaccine or a mAb formulation [29, 30, 31]. Although both potassium and sodium phosphate salts were reported to exert similar stabilizing effects [37], the former were preferred to reduce the hygroscopicity of the resulting powder. In fact, sodium salts are renowned for attracting more water with respect to potassium salts, which would be detrimental to the preservation of protein activity during storage [38]. Moreover, two bulking agents, trehalose and mannitol, were used to protect the serum from potential heat-induced damage due to the spray-drying procedure [39, 40]. These two excipients were already reported as effective in preserving the physical stability of proteins, in particular when bovine serum albumin was used as a model protein [41]. In this case, since the hyperimmune serum was not purified but was formulated as a whole, human serum albumin (HSA), being the main protein component of serum, was also present. HSA is expected to positively contribute to the interaction with excipients as well as to the protection of Igs, as previously reported, among many, for cytokines, mAbs, and a measles vaccine, probably by a surfactant-like effect [37, 42].

No further components commonly used in nasal formulations, such as for example penetration enhancers, were added, not only because the list of excipients admitted for inhalation is short [43], but also because the formulation was intended to act locally. Thus, Igs absorption should not be necessary, since their neutralizing activity should be exerted on the airway surface, by binding to viral particles and by immobilizing them in the mucus through a process defined as “muco-trapping” [11, 44]. This is due to the ability of the Fc domain of IgGs to crosslink with mucins, immobilizing viruses in a mucin mesh and favouring their elimination by mucociliary clearance.

The two powders, HS-T and HS-M were redissolved and tested to assess the preservation of their ability to bind a recombinant version of the Spike protein and to neutralize pseudoviral preparations corresponding to different SARS-CoV-2 variants of concerns, with respect to unformulated serum. For both formulations, the selected drying conditions slightly reduced the effectiveness of serum to neutralize all assayed pseudoviruses, nonetheless, the neutralizing ability was safeguarded. Thus, in view of a nasal administration, powders were characterized and tested for their physicochemical features and aerodynamic behaviour. Particle size analysis evidenced a monomodal distribution of HS-T (Dv_50_ 4.90 µm) and a bimodal distribution for HS-M (Dv_50_ 10.01 µm): both powders appeared adequate for inhalation. Apart from size, SEM analysis evidenced different morphologies for the two formulations, with HS-T appearing as fused smoother and more round particles, while HS-M looked like aggregated particles, partly explaining the bimodal distribution observed by particle size analysis. The appearance of these particles was quite different from those obtained by Chen et al*.* [41] after drying BSA as a model protein: those powders were composed of raisin-like wrinkled particles, independent of the excipient used. We ascribed such difference to the presence of phosphate salts in our formulations, which were added to better preserve the stability of Igs in solution and have been already reported to interfere with mannitol crystallization upon spray-drying of a recombinant monoclonal antibody [40]. DSC analyses on samples suggest the amorphous nature of HS-T and the presence of crystals in HS-M ascribable to mannitol.

Unfortunately, the limited volume of serum provided by the donor did not allow us to test more excipients or more combinations, to optimize the the formulation. Nevertheless, our data provide a clear proof-of-concept of the feasibility of the approach.

To exert their neutralizing role in the lungs, powders must deposit efficiently in the airways. Their aerodynamic performance was assessed in vitro by aerosolizing the powder with a nasal insufflator. The emitted powders were collected in an NGI apparatus equipped with an expansion chamber simulating the nasal cavity. The emitted dose of proteins and the amount of proteins impacting the expansion chamber were comparable for the two formulations, but their relative distribution in different portions of the airways was different. HS-M powder reached mainly the upper portion of the tracheobronchial region, while HS-T could reach to a higher extent the lower airways. The formulations could be adequate for a dual-targeting administration to the airways, both to the upper and lower airways, also considering the moment of administration with respect to the time that occurred since the contact with the virus. The moment of administration can be critical for preventing the development of symptoms or the progression to a severe disease. This could also influence the dose needed: infections are typically initiated in the upper respiratory tract with low titres of viruses, requiring a low dose of Abs; later, apical shedding of the virus, due to the destruction of infected cells, would require higher doses of antibodies, to prevent the stepwise propagation of the infection through the low respiratory tract and eventually the deep lung. The level of neutralizing activity required to protect against infection in a natural situation is unknown and this makes difficult the determination of a dose to be administered. In general, monoclonal antibodies reported as having an excellent neutralization ability have an IC50 below 15 ng/mL, and a near complete neutralization of viruses requires a 5–10 times higher concentration [8]. If dealing, such as in this case, with a polyclonal serum from a single donor, or in perspective from a pool of donors, the neutralizing ability should be determined as a result of the clonal selection of B-lymphocytes after exposure to antigens. For these reasons, instruments, such as assays to profile and compare polyclonal sera will be necessary [27, 45, 46] to get closer to the definition of a potential dose to administer, that will be anyway much lower than the one supposed to be administered parenterally [8].

## Conclusions

The serum of a subject immunized against SARS-CoV-2 can be formulated as a dry powder for nasal administration in the airways. The formulation and drying process only moderately reduce the neutralizing ability of the serum. Trehalose is a more promising bulking agent leading to higher yields in spray drying, better preservation of neutralizing ability as well as better aerosolization performance. Therefore, this approach can constitute an effective strategy to provide broad coverage and protection against SARS-CoV-2 and in general against viruses affecting the lungs, targeting at the same time the upper and lower airways. According to blood availability from donors, pools of hyperimmune sera could be rapidly formulated and administered, providing a simultaneous and timely neutralization of emerging viral variants. The same strategy could also be applied to purified sera, for example, enriched in IgAs, that are mainly involved in mucosal immunity.

## Supplementary Information

Below is the link to the electronic supplementary material.

Supplementary file1 (DOCX 1.37 MB)

Supplementary file2 (PPTX 441 KB)

Supplementary file3 (PPTX 441 KB)

Supplementary file4 (PPTX 441 KB)

Supplementary file5 (PPTX 441 KB)

## Data Availability

The datasets generated during and/or analysed during the current study are available from the corresponding author upon reasonable request.
