# Supplementary material for: Dry powder formulations of hyperimmune serum
Source: Drug Deliv Transl Res. 2024 Jul 31;15(4):1330–41. doi: 10.1007/s13346-024-01678-8 (PMC11870897; doi:10.1007/s13346-024-01678-8)
Supplement: Supplementary file 1 — Supplementary file1 (DOCX 1.37 MB) [file 13346_2024_1678_MOESM1_ESM.docx]

**Dry powder formulations of hyperimmune serum**

Annalisa Bianchera^1,2^, Gaetano Donofrio^2,3^, Fabio Sonvico^1,2^ and Ruggero Bettini^1,2^*

**Supplementary material**

**Table S1**. Particle size distribution of human serum-containing powders formulated with trehalose (FBS-T) or mannitol (FBS-M) as bulking agents.

| **Formulation** | **D_V(10)_ μm** | **D_V(50)_ μm** | **D_V(90)_ μm** |
| --- | --- | --- | --- |
| FBS-T | 2.44 ± 0.02 | 12.76 ± 0.13 | 26.06 ± 0.85 |
| FBS-M | 2.52 ± 0.08 | 8.15 ± 0.19 | 17.03 ± 40.91 |

a

b

**Figure S1** Particle size distribution as volume frequency (blue bars) and cumulative volume distribution (red line) of (a) FBS-T and (b) FBS-M.


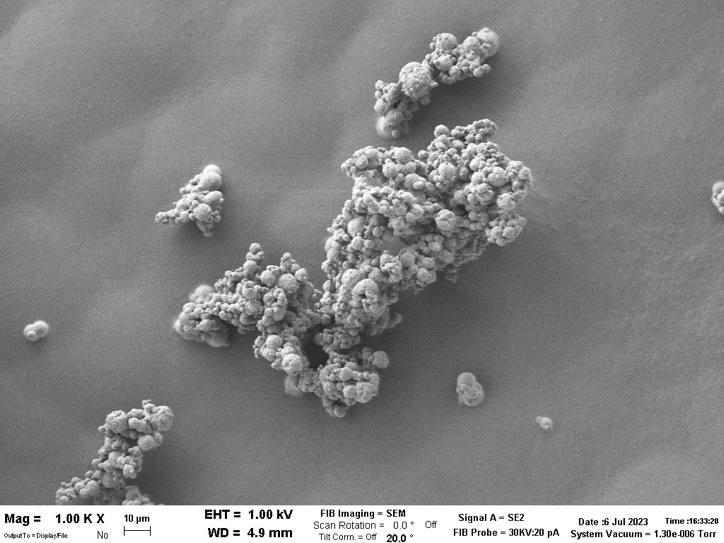

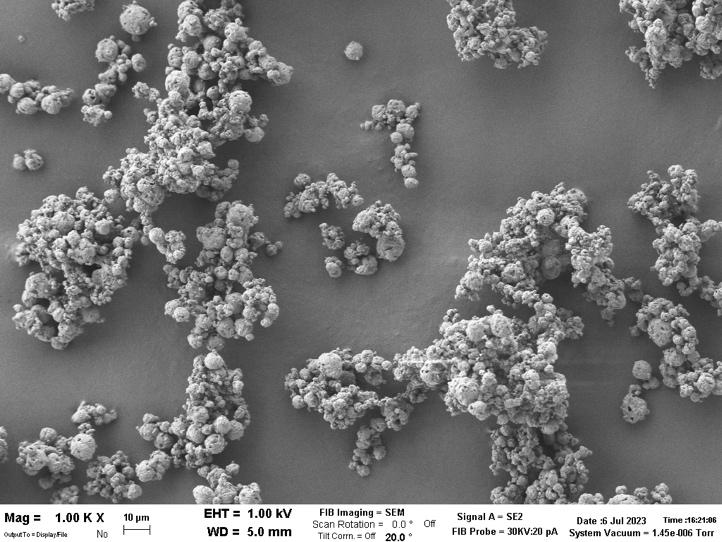

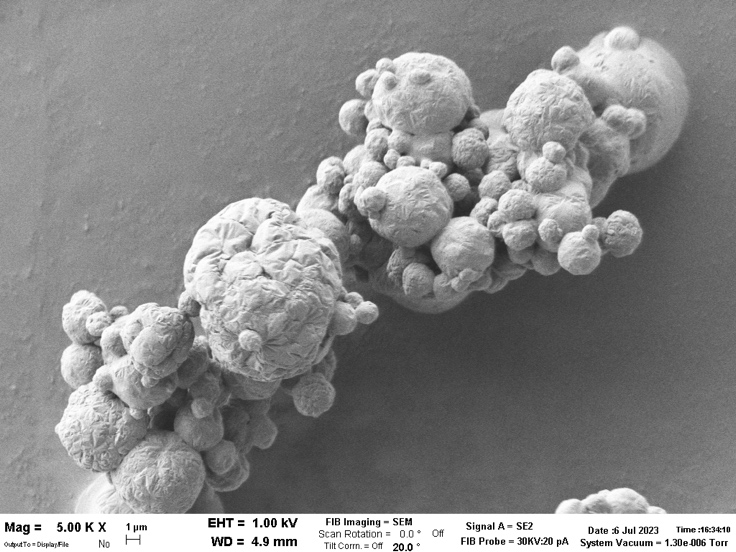

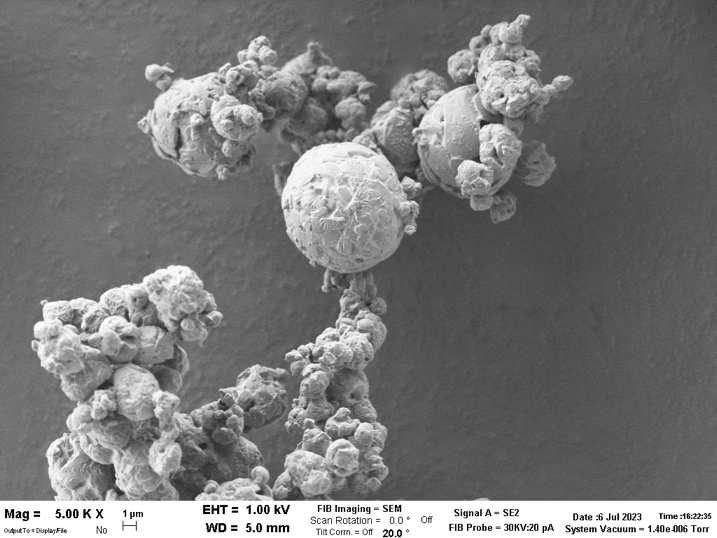


**c**

**d**

**b**

**a**

**Figure S2** SEM images of FBS-T (a-c) and FBS-M (b-d), magnification 1000 X (a-b) and 5000 X (c-d). White bars in the bottom right correspond to 10 µm in length.

**
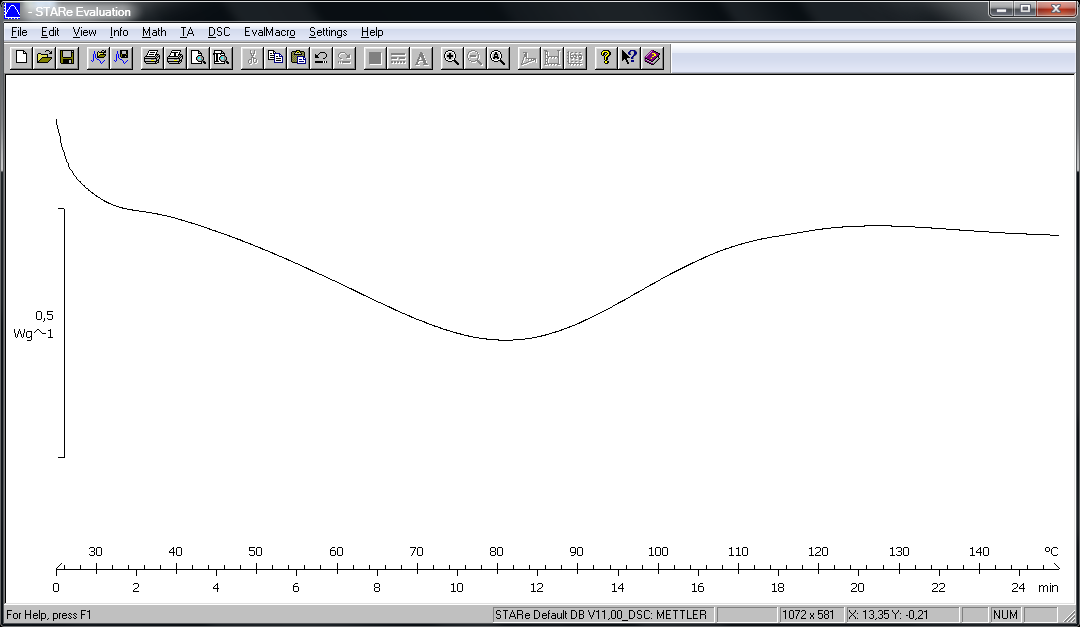

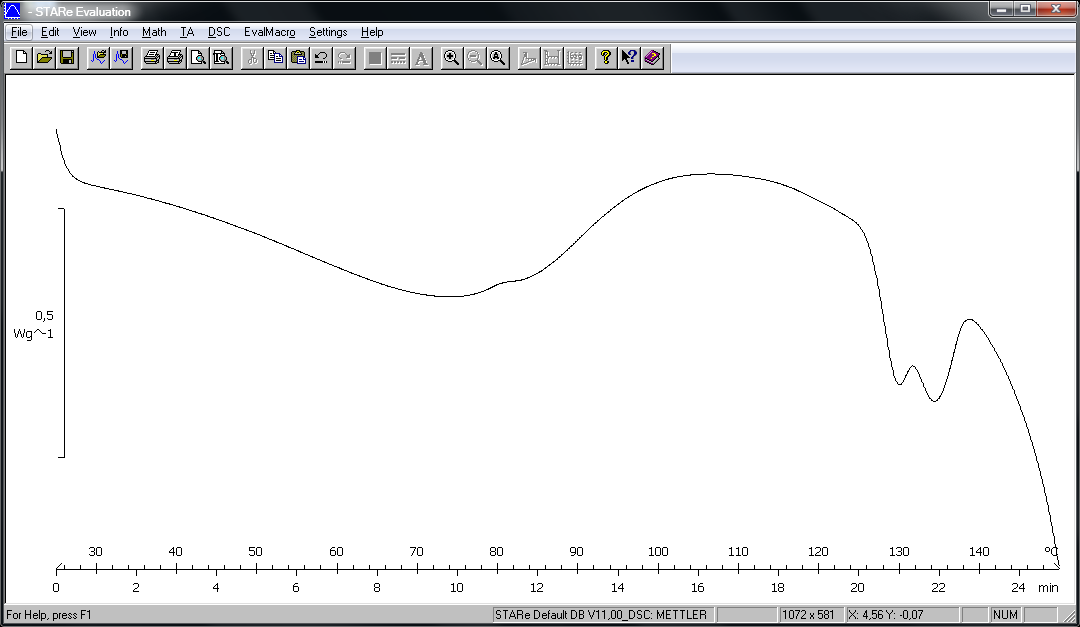
**

**Figure S3** DSC analysis of FBS-T (a) and FBS-M (b)

**Table S2.** Distribution in the NGI of hyperimmune serum formulated with trehalose (FBS-T) or mannitol (FBS-M) as bulking agents

|  | **FBS-T** | **FBS-M** |
| --- | --- | --- |
| Emitted dose of protein (mg) | 2.3 ± 0.8 | 2.0 ± 0.3 |
| Nasal dose of protein (mg) | 1.2 ± 0.3 | 1.6 ± 0.3 |
| Trachea + lung dose of protein (mg) | 0.5 ± 0.4 | 0.3 ± 0.3 |


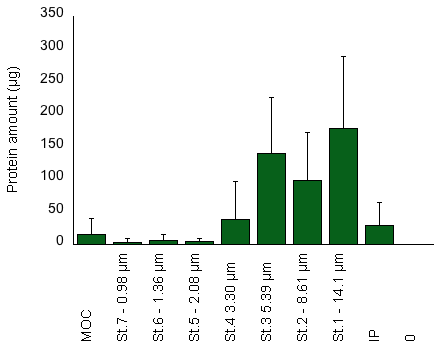

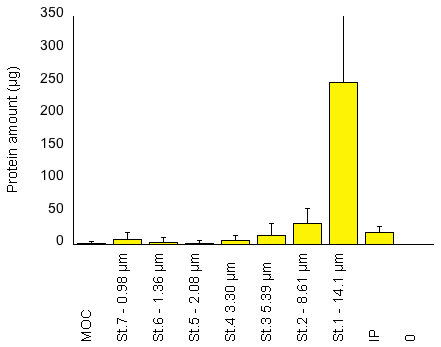


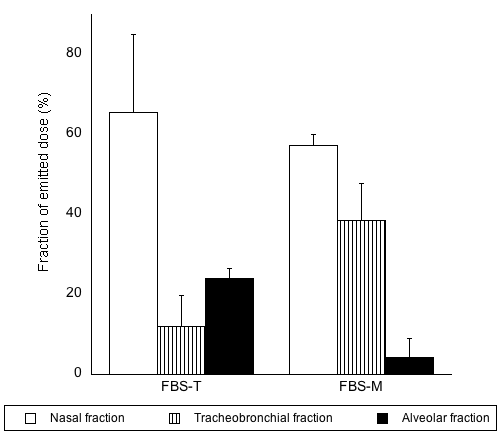


**Figure S4** Aerodynamic distribution of FBS-T (panel a) and FBS-M (panel b) in the stages of NGI. Fractions of formulations FBS-T and FBS-M supposed to reach the nose (empty column), tracheobronchial area (striped column), and alveoli (full column) after nasal inhalation (panel c). The bars represent the standard deviation (n=3)
